# JunD/HBZ enhances HBZ enhances HTLV-1 antisense transcription

**DOI:** 10.1186/1742-4690-8-S1-A135

**Published:** 2011-06-06

**Authors:** Jean-Marie Peloponese, Isabelle Lemasson, Benoit Barbeau, Jean-Michel Mesnard

**Affiliations:** 1 Centre d’études d’agents Pathogènes et Biotechnologies pour la Santé (CPBS), CNRS/UM1/UM2 UMR 5236, Montpellier, France; 2 East Carolina University, Department of Microbiology and Immunology, Greenville, North Carolina, 27834, USA; 3 Université du Québec à Montréal, Département des sciences biologiques, Montréal (Québec) Canada, H2X 3X8

## 

Human T-cell leukemia virus type 1 (HTLV-1) is an oncogenic retrovirus etiologically causal of adult T-cell leukemia (ATL). ATL is a highly virulent cancer that is resistant to chemotherapeutic treatments. To understand this disease better, it is important to comprehend how HTLV-1 promotes cellular growth and survival. The human T-cell leukemia virus type 1 (HTLV-1) basic leucine zipper factor (HBZ) gene is encoded by the minus strand of the HTLV-1 provirus and transcribed from the 3' long terminal repeat (LTR). HBZ expression promotes proliferation and survival of HTLV-1 infected cells in vivo. Although previous reports have been aimed at understanding the potential role of HBZ in HTLV-1 pathogenesis, little is known as to how this viral gene is regulated in ATL cells. Here using our K30-3’asLuc reporter construct, we show that HBZ protein upregulates antisense transcription. Generation of stable clones in 293T cells confirm that HBZ stimulates antisense transcription from the 3’ LTR through its interaction with JunD, an AP-1 protein, and its action on the Sp1 binding site located in the 3’LTR. Our results suggest that in vivo inhibition of JunD could be a possible new therapeutic strategy in ATL treatment.

